# Surge in Measles Cases in Italy from August 2023 to January 2025: Characteristics of Cases and Public Health Relevance

**DOI:** 10.3390/vaccines13070663

**Published:** 2025-06-20

**Authors:** Antonietta Filia, Martina Del Manso, Daniele Petrone, Fabio Magurano, Silvia Gioacchini, Patrizio Pezzotti, Anna Teresa Palamara, Antonino Bella

**Affiliations:** Department of Infectious Diseases, National Health Institute, 00161 Rome, Italy; martina.delmanso@iss.it (M.D.M.); daniele.petrone@iss.it (D.P.); fabio.magurano@iss.it (F.M.); silvia.gioacchini@iss.it (S.G.); patrizio.pezzotti@iss.it (P.P.); annateresa.palamara@iss.it (A.T.P.); antonino.bella@iss.it (A.B.)

**Keywords:** measles, outbreak, Europe, Italy, immunity gaps

## Abstract

**Background/Objectives:** A resurgence of measles has been observed in Europe and worldwide since 2023. The aim of this article is to describe characteristics of cases reported to the Italian national measles surveillance system and discuss reasons for the outbreak and its public health relevance. **Methods**: We analyzed measles cases reported to the Italian national measles surveillance system with a symptom onset from August 2023 to January 2025. **Results**: Overall, 1164 cases were reported, of which 1065 (91.5%) were laboratory confirmed. The median age was 30 years, but the highest incidence was in children under one year of age. Transmission occurred mainly in families and nosocomial settings. Multiple importations occurred during 2023, which initially led to localized outbreaks with limited spread but was subsequently followed by increasing local transmission in 2024. **Conclusions**: These findings suggest lingering immunity gaps in children and adults in Italy, highlighting the potential severity of measles and the ease with which measles crosses country borders. Besides improving routine immunization coverage, targeted vaccination opportunities should be provided to susceptible population groups. Communication activities are also needed to increase awareness about disease severity, especially among young adults. In view of the upcoming travel season, travel clinics should encourage persons without evidence of measles immunity to be vaccinated before any international travel.

## 1. Introduction

Measles is a highly infectious viral rash illness that is preventable by vaccination and that can cause severe complications, including pneumonia, acute encephalitis, and even death [1,2]. The World Health Organization (WHO) recommends that all countries include two doses of the measles-containing vaccine in their national immunization programs [3]. Since 1974, the measles vaccine has saved an estimated 93.7 million lives [4]. Measles elimination requires ≥95% two-dose coverage supported by high-quality surveillance [5,6]. Although 82 countries worldwide, and briefly, the entire region of the Americas, have achieved and verified elimination, no WHO region has managed to sustain it, highlighting how quickly immunity gaps allow endemic transmission to re-emerge [5,7].

In recent years, there has been a worldwide resurgence of measles, following a marked decline in the number of reported cases during the COVID-19 pandemic [8,9,10,11]. The WHO and U.S. Centers for Disease Control and Prevention estimate that there were 10.3 million cases globally in 2023, a 20% increase from 2022. The resurgence is caused by suboptimal global immunization coverage [8]. Measles vaccination coverage indicators worsened globally from 2019 to 2023, including the percentage of countries with two-dose measles vaccination coverage above or equal to 95% and the number of zero-dose children [12]. The estimated global vaccination coverage in 2023 was 83% for the first dose and only 74% for the second dose [8].

In the WHO European region, measles cases in 2024 more than doubled compared to 2023, reaching over 127,000 measles cases (including 38 deaths) detected in 49 of 53 countries in 2024, the highest number of cases since 1997 [10,11,12]. Most cases were unvaccinated, and all age groups were affected, though wide differences have been observed in age distribution between countries. Over 40% of cases were in children under five years of age [12]. Romania reported the highest number of cases in the region for 2024, with 30,692 cases, followed by Kazakhstan (28,147 cases) and the Russian Federation (22,076 cases), with Italy being among the 10 countries that reported the highest number of cases in the region in 2024 (1057 cases) [12].

The resurgence of measles in the WHO European region has been linked to disruptions in measles vaccination coverage, especially during the COVID-19 pandemic [5,10,11,13]. MCV coverage rates at the regional level in 2023 were 95% for the first dose and 91% for the second dose, with wide differences across and within countries [14].

In Italy, the measles vaccination first became available in the late 1970s, when tens of thousands of measles cases were being reported each year. However, uptake was initially very low. The vaccination became more widely available with the introduction of the combined measles–mumps–rubella (MMR) vaccine in the early 1990s [9]. Only one dose was recommended until 2003, when Italy implemented its first measles elimination plan. Increasing two-dose MMR uptake led to a gradual fall in incidence and a shifting of the median age of cases (from 18 years in 2010 to 30 years in 2019). A large outbreak occurred from 2017 to 2019, followed by a decline from March 2020 to July 2023 (134 cases in 2020, 26 in 2021, 14 in 2022, and 43 in 2023) [9]. Italy interrupted the endemic transmission of measles for at least 24 months in 2023, although it has not yet been verified by the European Regional Verification Committee to have reached the elimination target (which requires evidence of interruption of endemic transmission for a period of at least three years) [15,16]. Case notifications began rising again in August 2023, marking the onset of a new resurgence. This article aims to describe measles cases reported to the Italian national measles surveillance system with a symptom onset from August 2023 to January 2025 and discuss reasons for the outbreak and its public health relevance.

## 2. Materials and Methods

### 2.1. Data

We analyzed data on measles cases reported to the Italian national integrated measles and rubella surveillance system. The system is case-based, and all of Italy’s 21 administrative regions/autonomous provinces (APs) participated [17]. Physicians are required to report suspected measles cases to local health authorities (LHA) within 12 h of suspecting the diagnosis. For each reported case, local health authorities conduct an epidemiological investigation and collect biological specimens for laboratory confirmation. They then send a standardized measles notification form to their respective regional health authorities (RHAs) within 24 h. RHA input the data into a web-based platform managed by the Department of Infectious Diseases, Epidemiology, Biostatistics, and Mathematical Modeling Unit of the Italian National Health Institute (Istituto Superiore di Sanità-ISS). In some regions, local health authorities input the data directly into the web-based system by reporting cases through the platform.

For every notified case, complete epidemiological data is collected, including the following: date of birth; place of residence; healthcare worker status; clinical manifestations; vaccination history (status, number of doses, and date of last dose); laboratory results; contact with confirmed measles cases; travel history for Italian residents (and date of arrival for non-residents); transmission setting; complications; hospitalizations; and any emergency department consultations.

Cases are classified according to the European Commission case definitions [18] into possible, probable, or confirmed cases. A possible case is a person who meets the clinical criteria (fever, generalized maculopapular rash, and one of cough, coryza, or conjunctivitis) but lacks an epidemiological link or laboratory confirmation of disease. A probable case meets the clinical criteria and has an epidemiological link to a confirmed measles case within 7 to 18 days prior to rash onset. A confirmed case is one that meets the clinical and laboratory criteria. The laboratory criteria consist of at least one of the following: presence of measles-specific IgM antibodies in serum; significant (fourfold) increase in IgG antibody titers in paired serological samples taken, respectively, in the acute and convalescent phases of illness; detection of measles RNA in saliva, urine, or serum through RT-PCR; or isolation of the measles virus in a biological specimen. Cases are also classified by importation status.

### 2.2. Statistical Analysis

The categorical variables were summarized using frequencies and percentages, while age was described using a median and a range. The national incidence rates were calculated per 1,000,000 inhabitants and plotted by age group (<1, 1–4, 5–14, 15–39, 40–64, and >64 years). At the regional level, the incidence rates were calculated per 1,000,000 inhabitants based on regional population data. All calculations used 2024 population estimates (updated as of 1 January 2024) from the Italian National Institute of Statistics (ISTAT), available at demo.istat.it. The statistical analyses were performed using Stata version 16.1 (StataCorp LLC, College Station, TX, USA).

## 3. Results

### 3.1. Number and Classification of Cases, Including Imported Cases

**Figure 1:**
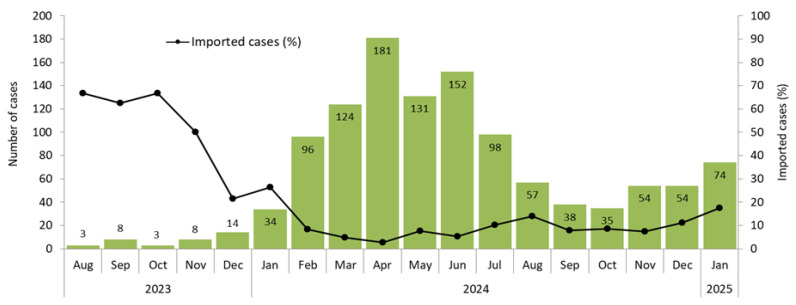
Number of reported measles cases and percentage of imported cases by month of onset of symptoms, Italy, August 2023 to January 2025.

Overall, 1164 cases were reported, of which 1065 (91.5%) were laboratory confirmed, 39 were probable (epidemiologically linked), and 60 were possible (clinically compatible) cases. Figure 1 shows the number of reported cases and the percentage of imported cases by month of symptom onset. The number of cases gradually increased from August 2023, reaching a peak of 181 cases reported in April 2024, then gradually declined until October 2024, when a new increasing trend was observed in 2025.

Overall, 109 cases (9.4%) were classified as imported. The percentage of imported cases progressively decreased from 66.7% in August 2023 to 2.8% in April 2024 and then increased to 17.6% in January 2025 (Figure 1). A total of 16 out of 36 cases (44.4%) reported from August to December 2023 were imported, and an additional 8 (22.2%) were import-related. In 2024, the percentage of imported and imported-related cases dropped to 7.6% (80/1054) and 1.6% (17/1054), respectively. The country of origin is known for 94 of 109 imported cases: 26 (27.6%) acquired the infection in Romania, and an additional 32 cases (34.1%) were imported from 15 other countries of the WHO European region. The remaining 36 cases (38.3%) were imported from various countries, mainly in southeast Asia and Africa (15 imported from Morocco).

### 3.2. Geographic Distribution of Cases

**Figure 2:**
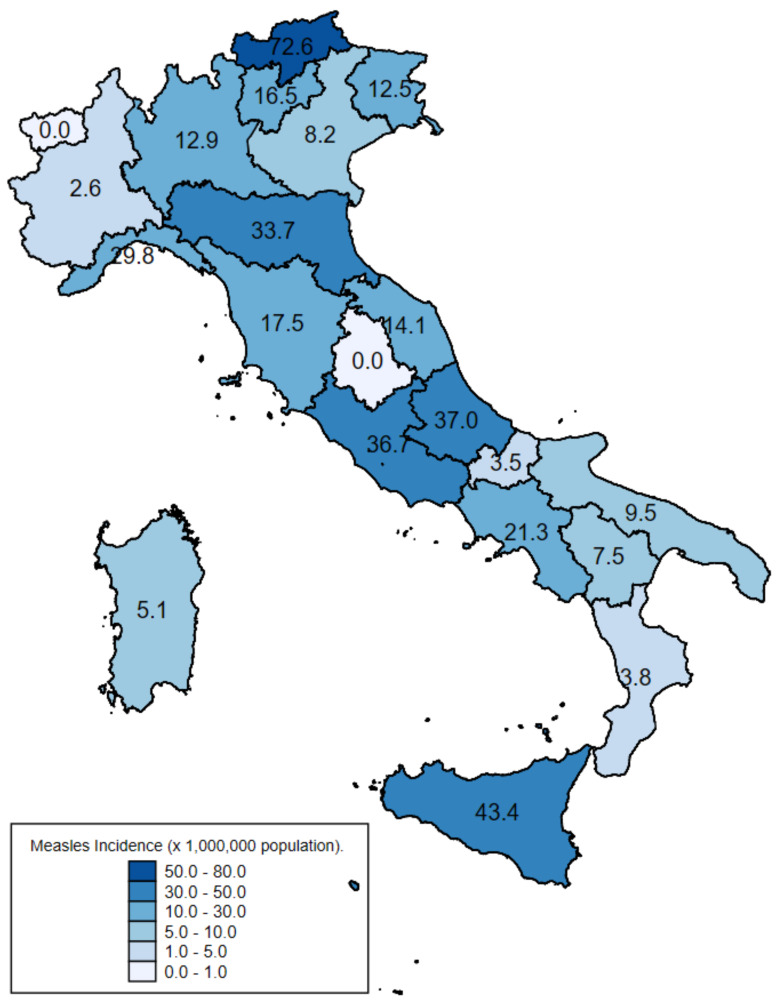
Incidence of measles notifications per million population by administrative region. Italy, August 2023 to January 2025.

A total of 19 out of 21 regions/APs reported cases, but 75.6% of cases were reported from 6 regions: Lombardy and Emilia-Romagna in northern Italy, Tuscany and Lazio in central Italy, and Campania and Sicily in southern Italy (Figure 2). The highest incidence was observed in the AP Bolzano (northern Italy) and in Sicily (southern Italy), with 72.6 and 43.4 cases per million population, respectively (Figure 2).

### 3.3. Demographic and Clinical Characteristics of Cases

**Figure 3:**
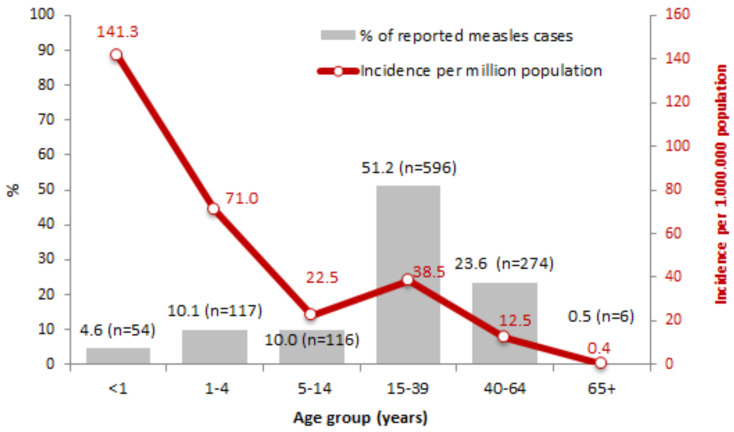
Number of measles cases, percentage distribution, and incidence per million by age group. Italy, August 2023 to January 2025 (*n* = 1164).

The median age was 30 years (range: 0–73 years), and 53.2% were male. Figure 3 shows the number of reported cases, the percentage distribution, and the incidences by age group. Over half of the cases (51.2%) were aged 15 to 39 years, and an additional 24.1% were over 40 years of age. However, the highest incidence was observed in infants (54 cases, 141.3/million age-specific population), followed by children aged 1–4 years.

### 3.4. Vaccination Status

The vaccination status was known for 1088 cases (93.5%). Among the cases eligible for vaccination (persons > 12 months of age, *n* = 1034), 89.9% were unvaccinated, 5.6% (*n* = 61) had received only one dose, and 3.3% (*n* = 36) were vaccinated with two doses. The number of doses is unknown for seven cases.

### 3.5. Complications and Hospital Admissions

A total of 403 cases (34.6%) reported at least one complication, the most frequent being hepatitis (*n* = 168; 14.4% of cases), followed by diarrhea (*n* = 131; 11.3%) and pneumonia (*n* = 125; 10.7%). Other complications included keratoconjunctivitis (*n* = 95, 8.2%), thrombocytopenia (*n* = 30; 2.6%), and otitis media (*n* = 29; 2.5%). Of note, two cases of acute measles encephalitis were reported, both in unvaccinated adults. One child (0.1%) developed convulsions. No deaths were reported. Overall, 585 of 1156 cases (50.6%), for whom information is available, required hospital admission. An additional 211 cases (18.3%) received outpatient care in a hospital emergency department.

### 3.6. Transmission Settings

The transmission setting is known for 535 cases (46.0%). The main transmission setting was household transmission (42.6% of cases), followed by transmission in nosocomial or healthcare settings (20%). Other transmission settings are shown in Table 1. Eighty-seven cases (7.5%) were healthcare workers (HCWs), defined as any hospital or other healthcare staff having regular contact with patients. Cases among HCWs (median age of 33 years, range: 23–56 years) were reported from 14 regions; 80.2% were unvaccinated.

### 3.7. Genotyping

Genotype results are available for 658 cases (61.8%). D8 was identified in 628 cases (95.4%), and B3 was identified in 30 cases.

### 3.8. Public Health Measures

Regional health authorities issued circulars or guidelines to inform HCWs and other stakeholders of the increased incidence and to recommend outbreak control measures. Circular/guideline recommendations included intensification of surveillance activities, isolation of cases, vaccination of susceptible contacts, administration of immunoglobulins to contacts at high risk of complications, supplementary vaccination activities, isolation and infection-control protocols in healthcare settings, and communication activities aimed at HCWs and the population. Some also recommended limiting referral of cases to hospitals only to cases presenting symptoms or signs of a complication to prevent nosocomial transmission, verifying the immunity status of all hospital HCWs, and vaccinating susceptible HCWs. The Italian National Health Institute (Istituto Superiore di Sanità-ISS) publishes monthly reports describing the measles situation in the country by region and providing an analysis of the data [19].

## 4. Discussion

The described surge in measles cases in Italy followed multiple importations of the virus in 2023, mainly by unvaccinated travelers returning from areas with high circulation of the virus, highlighting the ease with which measles crosses cross-country borders. Cases were imported mainly from other European countries; in fact, increased measles activity has been reported across Europe during the same time period, including in countries of the EU/EEA [20]. The importations initially led to small, localized outbreaks that year, but, by 2024, most cases were due to local transmission, suggesting the existence of immunity gaps in the population. Whether endemic transmission has been re-established in Italy will be evaluated later in 2025 when 2024 epidemiological and genotyping data are reviewed by the European Regional Verification Commission for measles and rubella elimination (RVC).

Most of the reported cases occurred in unvaccinated or incompletely vaccinated people, and over half of the cases were adolescents and young adults, suggesting that there continue to be large shares of susceptible people in this specific population [21]. The age distribution of cases is different in Italy compared to that of the EU/EEA, where only 26% of cases reported in 2024 were 15 years of age or older (the median age of the cases was 5 years) [20]. However, the age distribution in the EU/EEA in 2024 is driven mainly by the high number of cases in Romania, where most cases were reported among the 1–4-year-old age group [20]. Besides Italy, several other countries in the EU/EEA reported a larger percentage of cases in adults above 30 years of age (France: 28.4%, Poland: 34.4%, Spain: 38.5%). The different age patterns likely reflect long-standing differences in vaccine uptake and gaps in catch-up efforts. Romania has recently experienced a substantial fall in measles coverage among young children, favoring transmission at younger ages, whereas countries with higher current childhood coverage but missed cohorts in earlier years now face greater susceptibility among older age groups [20].

A recently published seroepidemiological study was carried out in 2019–2020 among 3746 subjects aged 6–64 years residing in 13 Italian regions [22]. The study showed an overall seroprevalence of measles-specific IgG antibodies of 91.2%, which is below the 95% target but significantly higher than the seroprevalence reported in previous surveys performed in 1996–1997 (83.1%) and 2003–2004 (77.2%) using the same sampling methodology and laboratory tests. However, differences were observed across the age groups. The seroprevalence was highest in children aged 6–9 years (94.2 %), declined in the 10–39-year-old cohorts (range: 89.3–89.7%), and rose again among adults ≥ 40 years (97.6 %). The lower seroprevalence among adolescents and young adults reflects the age distribution of reported cases. Similar seroprevalence profiles, with immunity gaps in young adults, have been observed in other European countries that have recently reported outbreaks, such as Austria, Belgium, and Germany [20,23,24,25]. Such profiles reflect the suboptimal vaccination coverages in the first years after the routine measles vaccination was introduced [23,24,25].

Since 2017, the MMR vaccination has been mandatory in Italy up to 16 years of age. Proof of vaccination is required to attend nursery and kindergarten school but not for elementary or middle school enrollment (financial sanctions are applied). MMR is also offered free of charge to adolescents and adults up to 59 years of age who have no documented immunity to measles. However, more vaccination opportunities should be provided to the latter population group, especially during other occasions of contact with the healthcare system, including in travel clinics, where those without evidence of immunity can be vaccinated before any international travel. This is especially important ahead of the peak holiday season. Targeted communication activities are likewise essential to increase awareness of measles among young adults, and its potential severity.

The highest notification rates, as in the rest of the EU/EEA, were in small children below five years of age. Cases in infants and small children are especially worrisome as they are at a high risk of severe illness and death and of developing subacute sclerosing panencephalitis [26]. Infants below one year of age are too young to be vaccinated and, therefore, depend on maternal antibodies and population immunity to be protected. To ensure their protection in an outbreak setting, the WHO Regional Office for Europe recommends the first vaccine dose may be moved up from 12 months to 9 months of age and, in some circumstances, can be given as early as 6 months [6]. Children who receive a dose of MMR vaccine before 12 months of age should receive two additional doses of vaccine according to the national immunization schedule.

Disruptions in vaccine uptake in Italy during the COVID-19 pandemic have likely led to an accumulation of susceptible children in recent years. First-dose measles vaccine uptake among children at 24 months of age decreased by 1.8 percentage points from 94.5% in 2019 to 92.7% in 2020 [27]. Second-dose coverage also decreased during the same time period from 87.6% to 85.8% [27]. Since then, first-dose coverage has gradually returned to prepandemic levels (94.6% at 24 months of age in 2023) but remains suboptimal (<95%), while second-dose uptake at 5–6 years of age has continued its downward trend and was 84.8% in 2023. There are also wide variations at the subnational level (range 83.8–97.3% for the first dose and 71–94.2% for the second dose) [27]. The two regions that did not report cases during the study period are small regions, and both had a two-dose uptake above 90% in 2023. On the other hand, the two regions/APs with the highest incidence both had a low two-dose uptake (74.8% and 71.0%, respectively). The reasons for suboptimal uptake should be verified and addressed, including identifying any existing immunization inequities, as recommended by the WHO [28].

As in previously reported outbreaks in Italy, cases were reported among healthcare workers (HCWs), and the nosocomial setting was an important transmission setting [17,21]. This highlights the importance of HCW vaccination against measles, as recommended by the WHO and ECDC [10,29]. HCWs are at a higher risk of acquiring measles and of transmitting it to vulnerable patients [10,29]. However, the MMR vaccination is generally not required for employment as an HCW in Italy. 

Alongside improving vaccination uptake among HCWs, clinicians must maintain a high index of suspicion for measles in patients with febrile rash illness so that cases may be diagnosed and reported in a timely manner and infection control guidelines implemented in all healthcare settings. Finally, given that HCWs are the most trusted source of information for individuals who have questions about vaccines, ensuring that they are both protected and able to confidently advocate for vaccination is essential to curbing nosocomial transmission and safeguarding the public. The WHO urges countries to improve the availability and use of high-quality, evidence-based information aimed at healthcare professionals and the public on vaccines and the diseases they prevent [5].

Sustained efforts are needed to protect gains toward measles elimination [5]. As recommended by the WHO Regional Office for Europe in the recently published integrated guidance document on surveillance, outbreak response, and the verification of elimination in the European region, tailored approaches are necessary to achieve and maintain high sustained coverage at all subnational levels and to close existing immunity gaps [5,6]. Countries are also urged to strengthen their surveillance systems and ensure adequate outbreak preparedness and response [5,6].

### Study Limitations

Our analysis relies on cases reported to the Italian National Integrated Measles and Rubella Surveillance System. While this is the official source for monitoring measles in Italy, several inherent weaknesses of any passive surveillance system should be considered when interpreting the results. Some measles cases, especially milder cases, may not be diagnosed or formally reported, so surveillance data almost certainly underestimates the true burden of disease. Also, although key epidemiological variables, such as vaccination status, transmission setting, complications, and hospitalization, are included in the standard case-investigation form, the information is not always collected or entered into the web platform. This may lead to missing or miscoded values; for example, in our dataset, the probable transmission setting was available for fewer than 50% of the reported cases. Lastly, case records may sometimes be uploaded to the web platform with delay. For this analysis, we used the most up-to-date, consolidated dataset available at the time of extraction, which should minimize, though not entirely eliminate, bias due to late reporting. Despite these limitations, the national measles surveillance system remains the most comprehensive and authoritative dataset for assessing national measles trends, and the findings presented here should be viewed in that context.

## 5. Conclusions

Despite considerable progress toward interrupting endemic measles transmission, our findings suggest persistent immunity gaps among both children and adults in Italy. These shortfalls reflect the long-standing suboptimal vaccination coverage, exacerbated by reduced uptake during the COVID-19 pandemic. Various countries in Europe face similar challenges.

To protect recent gains achieved toward the interruption of endemic measles transmission in Italy, the strategies recommended by WHO and ECDC need to be fully implemented [5,6,12]. Timely administration of both first and second MMR doses is vital, in accordance with the national schedule for children. Catch-up activities are likewise needed to close immunity gaps among children and adults, including using every encounter with the health care system as an opportunity to verify measles immunity status and offer vaccination to persons lacking documented immunity [12].

## Figures and Tables

**Table 1:** Transmission settings (*n* = 535) for reported measles cases. Italy, August 2023 to January 2025.

Transmission Setting	*n*.	%
Household	228	42.6
Nosocomial/healthcare	107	20.0
Travel	94	17.6
Work (excluding healthcare settings)	47	8.8
School	30	5.6
Prisons	9	1.7
Public gathering events	11	2.0
Other	9	1.7
Total	535	100

## Data Availability

Aggregated data supporting reported results are available upon request. Individual-level data are unavailable due to privacy restrictions.
